# Characteristics Associated with Negative Interferon-γ Release Assay Results in Culture-Confirmed Tuberculosis Patients, Texas, USA, 2013–2015

**DOI:** 10.3201/eid2403.171633

**Published:** 2018-03

**Authors:** Duc T. Nguyen, Larry D. Teeter, Julie Graves, Edward A. Graviss

**Affiliations:** Houston Methodist Research Institute, Houston, Texas, USA (D.T. Nguyen, E.A. Graviss);; Forensic Research and Analysis, Portland, Oregon, USA (L.D. Teeter);; University of Medicine and Health Sciences, New York, New York, USA (J. Graves);; University of Texas School of Public Health, Houston (J. Graves)

**Keywords:** tuberculosis, IGRA, QuantiFERON, T-SPOT, T-SPOT.*TB*, culture, TB disease, LTBI, bacteria, mycobacteria, HIV/AIDS and other retroviruses, tuberculosis and other mycobacteria, *Mycobacterium tuberculosis*, diagnostics, epidemiology, interferon-γ release assay, United States, Texas, false-negative results

## Abstract

Interferon-γ release assays (IGRAs) are the preferred diagnostic test for tuberculosis (TB) infection in at-risk populations in developed countries. However, IGRAs have high false-negative rates in patients with TB disease. Population-based studies assessing the factors associated with negative IGRA results in TB patients have not been performed. Using statewide TB surveillance data of culture-confirmed TB patients in Texas, USA, during 2013–2015, we describe the patient characteristics and treatment outcomes associated with false-negative IGRA results. Among 2,854 TB patients, 1,527 (53.5%) had an IGRA result; 97.4% (1,487/1,527) of those had a positive (87.7%) or negative (12.3%) result. Older age, HIV co-infection, non-Hispanic white race/ethnicity, and being tested with T-SPOT.*TB* were associated with negative IGRA results. TB patients with negative IGRA results had a higher mortality, potentially due to delayed treatment. Healthcare providers should consider these risk factors when making decisions for patients with suspected TB and negative IGRA results and potentially provide treatment.

Interferon-γ release assays (IGRAs) are blood tests that measure immune reactivity to *Mycobacterium tuberculosis*–specific antigens and aid in the diagnosis of latent tuberculosis (TB) infection (LTBI). During the period of this study, 2 IGRAs, which have been approved by the US Food and Drug Administration, were commercially available for use in the United States: QuantiFERON-TB Gold In-Tube (QFT; QIAGEN, Germantown, MD, USA); and T-SPOT.*TB* (Oxford Immunotec, Inc., Marlborough, MA, USA) (*1*). In Texas, a state with one of the highest TB burdens in the United States, IGRAs have been used in targeted testing among persons at high risk for LTBI or at high risk of developing TB disease once infected with TB (*2*). Although IGRAs are not recommended to be used as rule-out tests for TB disease because of their inability to differentiate LTBI from TB disease and inconsistent sensitivity and specificity in different populations with TB disease (*3**–**7*), IGRAs are still preferred tools used by healthcare providers to identify LTBI in persons being evaluated for TB disease before biological confirmation. In the absence of other positive rapid diagnostic test results for TB disease, a negative IGRA result might inappropriately lower the clinical suspicion for TB and result in delayed treatment initiation. Previous studies have suggested that older age, underweight, HIV co-infection, extrapulmonary TB, and increased number of human leukocyte antigen DRB1*0701 alleles were associated with negative IGRA results (*8**–**11*). However, these studies had small sample sizes or were performed at a single center. In addition, little information is available on deaths associated with negative IGRA results in a population with confirmed TB. The main objectives of our population-based analysis were to identify the demographics, clinical characteristics, and patient outcomes associated with negative IGRA results in patients with culture-confirmed TB.

## Methods

We acquired deidentified TB surveillance data of patients with TB disease reported in the Texas National Electronic Disease Surveillance System (NEDSS) database during January 1, 2013–December 31, 2015. NEDSS is a TB surveillance data system used by all state jurisdictions within the United States to facilitate the electronic transfer of public health surveillance data from the healthcare system to public health departments (*12*). The downloaded data included sociodemographic, clinical, laboratory, and radiographic characteristics. We downloaded genotype information from the Centers for Disease Control and Prevention TB Genotyping Information Management System (*13*). We included for analysis only the TB patients who were confirmed TB positive by *M. tuberculosis* culture and had either a positive or negative IGRA result available. We excluded TB patients with negative culture results and those for whom specimens were not taken for TB culture (clinical TB patients), as well as those with unavailable or unknown IGRA types or indeterminate (for QFT) or failed (for T-SPOT.*TB*) IGRA results. We defined the diagnosis date as the specimen collection date of the first *M. tuberculosis*–positive culture and the time to TB treatment as the number of days from the diagnosis date to the date when the TB treatment started.

### Statistical Analysis

We reported demographic and clinical characteristics as medians and interquartile ranges (IQRs) for continuous variables and as numbers and percentages for categorical variables. We compared the differences between groups (e.g., IGRA-positive group vs IGRA-negative group) using the Kruskal-Wallis test for continuous variables and the χ^2^ test for categorical variables. We used logistic regression with the robust standard error option to obtain unbiased standard errors and calculated odds ratios (ORs) and 95% CIs to identify potential associations between negative IGRA results and demographic, clinical, or laboratory characteristics. We further investigated variables having a p value of <0.2 in the univariate logistic regression by multiple logistic regression modeling to identify characteristics significantly associated with negative IGRA results. We analyzed the survival during the 1-year period after TB diagnosis and stratified by IGRA type using the Kaplan-Meier method. We compared the survival differences between IGRA types using the log-rank test. We used Cox proportional hazards modeling to determine the risk factors associated with death during the 1-year period after TB diagnosis. We conducted variable selection for the multiple logistic regression and Cox proportional hazards models using the Bayesian model averaging method (*14**,**15*). In brief, we ran Stata’s Bayesian model averaging program to evaluate possible model sets for all variables having a p value of <0.2 in the univariate analysis, and Stata suggested good models that included the variables with a high posterior probability of being a risk factor. We used the likelihood ratio test to further reduce the model subsets and selected the best model on the basis of its small Bayesian information criterion. We also tested the selected models for the proportional hazard assumption and evaluated model discrimination using the Harrell C statistic. We performed all analyses with Stata version 14.2 (StataCorp LLC, College Station, TX, USA) and considered p values <0.05 statistically significant.

## Results

**Figure 1:**
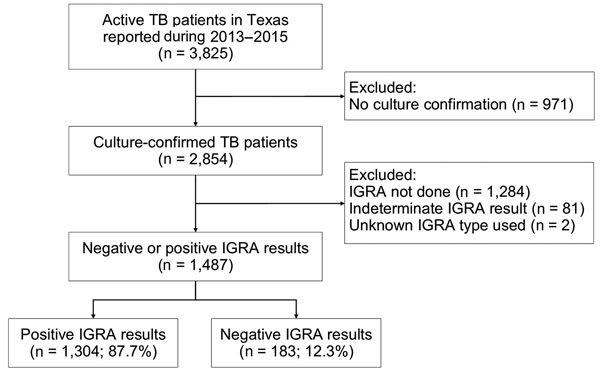
Flowchart showing selection of culture-confirmed TB patients with IGRA results, Texas, USA, 2013–2015. IGRA. IGRA, interferon-γ release assay; TB, tuberculosis.

Among the 3,825 patients with TB disease reported in Texas during 2013–2015, a total of 2,854 (74.6%) were confirmed positive by *M. tuberculosis* culture (Figure 1). Of the 2,854 patients with culture-confirmed TB, 1,527 (53.5%) had an IGRA result, with 97.4% (1,487/1,527) having either a positive (87.7%, 1,304/1,487) or negative (12.3%, 183/1,487) result. Of the 2,854 culture-confirmed TB patients, 1,367 (47.9%) were excluded from analysis, either because the IGRA was not done or reported or the IGRA was done but the result was indeterminate or the test type was not specified in the surveillance record (Figure 1). Compared with the patients included in analysis, the patients excluded from analysis were less likely to be Asian and more likely to be men, foreign-born persons, persons who used alcohol in excess or were injection drug users, persons with TB-related radiographic abnormalities, and persons who died (Technical Appendix Table).

The patients included in the study sample (N = 1,487) had a median age of 47 (IQR 30–61) years, and most were foreign-born (60.3%); 63.3% were men (Table 1). More than half (51.2%) of the population was Hispanic. Most of the patients had a chest radiograph consistent with TB disease (83.4%). A total of 105 (7.1%) TB patients died within 1 year of TB diagnosis (Table 1).

**Table 1:** Characteristics of 1,487 culture-confirmed TB patients with negative and positive IGRA results, Texas, USA, 2013–2015*

Characteristic	Value
Age, y, median (IQR)	47.0 (30.0–61.0)
Age >60 y	402 (27.0)
Sex	
M	942 (63.3)
F	545 (36.7)
Race/Ethnicity	
Non-Hispanic white	152 (10.2)
Black	275 (18.5)
Hispanic	762 (51.2)
Asian	288 (19.4)
Other	10 (0.7)
Foreign-born	897 (60.3)
Resident of long-term care facility	20 (1.3)
Homeless	100 (6.7)
Excess alcohol user	257 (17.3)
Injection drug user	38 (2.6)
Chest radiograph result	
Normal	160 (10.8)
Consistent with TB	1,240 (83.4)
Unknown/Not done	87 (5.9)
TB site	
Pulmonary	1,136 (76.4)
Extrapulmonary	196 (13.2)
Both	155 (10.4)
HIV status	
Negative	1,279 (86.0)
Positive	90 (6.1)
Unknown	118 (7.9)
MDR	13 (0.9)
Days from diagnosis to treatment, median (IQR)	3.0 (0–13.0)
Died within 1 year of TB diagnosis	105 (7.1)

*Values are no. (%) unless otherwise indicated. Percentages were calculated on the basis of the number of patients with data available. IGRA, interferon-γ release assay; IQR, interquartile range; MDR, multidrug resistance; TB, tuberculosis.

Compared with patients having positive IGRA results (median age 46 years), those having negative IGRA results were older (median age 55 years; p<0.001), typically >60 years of age (OR 2.13, 95% CI 1.55–2.93). Patients with negative results were also more likely than those with positive results to be non-Hispanic white (OR 2.50, 95% CI 1.66–3.77), US-born (OR 1.37, 95% CI 1.01–1.87), and HIV-infected (OR 2.23, 95% CI 1.32–3.78) (Table 2). Culture-positive, IGRA-negative patients were more likely than culture-positive, IGRA-positive patients to have been tested with the T-SPOT.*TB* assay (OR 1.41, 95% CI 1.03–1.94). Culture-positive, IGRA-negative patients had a significantly longer median time from diagnosis to treatment initiation (5 [IQR 0.5–26.0] days) than culture-positive, IGRA-positive patients (2 [IQR 0–12] days). The proportion of specific genotype clusters between patients having negative (48.6%) and positive (44.1%) IGRA results was not significantly different (p = 0.26). The *M. tuberculosis* genotype cluster G00010 was the most common found in study patients, with no significant difference found between the negative (3.3%) and positive (2.6%) IGRA groups (p = 0.60) (Table 2). Fewer IGRA-negative patients reported contact with an infectious TB patient during the previous 2 years (OR 0.28, 95% CI 0.09–0.90) and, therefore, were less likely to be epidemiologically linked to a TB patient (OR 0.46, 95% CI 0.22–0.96). Multivariate analysis results suggested that older age, non-Hispanic white race/ethnicity, HIV co-infection, being tested with the T-SPOT.*TB* assay, and longer time to TB treatment were significantly associated with having a negative IGRA result (Table 3). In stratified analyses by IGRA type, a negative QFT result was significantly associated with older age, non-Hispanic white race/ethnicity, and HIV co-infection, and a negative T-SPOT.*TB* result was significantly associated with older age, non-Hispanic white race/ethnicity, and longer time to TB treatment (Table 3).

**Table 2:** Crude association between negative IGRA results and culture-confirmed TB patient characteristics, Texas, USA, 2013–2015*

Variable	Positive IGRA, n = 1,304	Negative IGRA, n = 183	Crude OR (95% CI)	p value
Age, y, median (IQR)	46 (29–60)	55 (40–68)	1.02 (1.01–1.03)	<0.001
Age >60 y	326 (25.0)	76 (41.5)	2.13 (1.55–2.93)	<0.001
Sex				
F	479 (36.7)	66 (36.1)	Reference	
M	825 (63.3)	117 (63.9)	1.03 (0.75–1.42)	0.861
Non-Hispanic white	116 (8.9)	36 (19.7)	2.50 (1.66–3.77)	<0.001
US born	505 (38.7)	85 (46.4)	1.37 (1.01–1.87)	0.046
Homeless	85 (6.5)	15 (8.2)	1.28 (0.72–2.27)	0.40
Resident of correction institution	50 (3.8)	4 (2.2)	0.56 (0.20–1.57)	0.27
Resident of long-term care facility	18 (1.4)	2 (1.1)	0.79 (0.18–3.43)	0.75
Injection drug user	35 (2.7)	3 (1.6)	0.60 (0.18–1.99)	0.41
Excessive alcohol user	218 (16.7)	39 (21.3)	1.35 (0.92–1.98)	0.13
Contact with TB patient within past 2 y	73 (5.6)	3 (1.6)	0.28 (0.09–0.90)	0.03
Received TNF-α antagonist therapy	2 (0.2)	1 (0.5)	3.58 (0.32–39.64)	0.30
Solid organ transplant recipient	1 (0.1)	1 (0.5)	7.16 (0.45–114.96)	0.17
Diabetes	265 (20.3)	39 (21.3)	1.06 (0.73–1.55)	0.76
Chronic kidney disease	16 (1.2)	3 (1.6)	1.34 (0.39–4.65)	0.64
Immunosuppression	30 (2.3)	7 (3.8)	1.69 (0.73–3.90)	0.22
Previous TB	31 (2.4)	1 (0.5)	0.23 (0.03–1.66)	0.14
Chest radiograph result				
Normal	137 (10.5)	23 (12.6)	Reference	
Consistent with TB	1,092 (83.7)	148 (80.9)	0.81 (0.50–1.30)	0.38
Unknown/Not done	75 (5.8)	12 (6.6)	0.95 (0.45–2.02)	0.90
TB site				
Pulmonary	997 (76.5)	139 (76.0)	Reference	
Extrapulmonary	169 (13.0)	27 (14.8)	1.15 (0.74–1.79)	0.55
Both	138 (10.6)	17 (9.3)	0.88 (0.52–1.51)	0.65
HIV status				
Negative	1,134 (87.0)	145 (79.2)	Reference	
Positive	70 (5.4)	20 (10.9)	2.23 (1.32–3.78)	0.003
Unknown/Not done	100 (7.7)	18 (9.8)	1.41 (0.83–2.39)	0.21
Epidemiologically linked	118 (9.0)	8 (4.4)	0.46 (0.22–0.96)	0.04
Tested by T-SPOT.*TB*	436 (33.4)	76 (41.5)	1.41 (1.03–1.94)	0.03
IGRA sample collected after TB treatment initiated	415 (31.8)	74 (40.4)	1.45 (1.06–20)	0.02
MDR	12 (0.9)	1 (0.5)	0.59 (0.08–4.58)	0.62
Genotyped	1,260 (96.6)	175 (95.6)	0.76 (0.35–1.65)	0.49
East Asian family lineage	231 (17.7)	27 (14.8)	0.80 (0.52–1.24)	0.32
Clustered	555 (44.1)	85 (48.6)	1.20 (0.87–1.65)	0.26
GENType G00010	34 (2.6)	6 (3.3)	1.27 (0.52–3.06)	0.60
Days from diagnosis to treatment initiation, median (IQR)	2.0 (0–12.0)	5.0 (0.5–26.0)	1.00 (1.00–1.01)	0.04
Follow-up, mo, median (IQR)†	9.4 (7.3–10.7)	9.2 (7.0–10.7)	0.96 (0.92–1.01)	0.11
Year of diagnosis				
2013	373 (28.6)	56 (30.6)	Reference	
2014	428 (32.8)	53 (29.0)	0.82 (0.55–1.23)	0.35
2015	503 (38.6)	74 (40.4)	0.98 (0.68–1.42)	0.92

*Values are no. (%) unless otherwise indicated. GENType, tuberculosis genotype; IGRA, interferon-γ release assay; IQR, interquartile range; MDR, multidrug resistance; OR, odds ratio; TB, tuberculosis; TNF-α, tumor necrosis factor α. †From diagnosis date until the end of treatment or patient death, whichever came first.

**Table 3:** Multivariate analysis of association between negative IGRA results and culture-confirmed TB patient characteristics, by IGRA type, Texas, USA, 2013–2015*

Variable	All IGRA results, N = 1,338†			QFT only, n = 875†			T-SPOT.*TB* only, n = 463†	
aOR (95% CI)	p value		aOR (95% CI)	p value		aOR (95% CI)	p value
Age in years	1.02 (1.01–1.03)	<0.001		1.02 (1.01–1.04)	<0.001		1.02 (1.00–1.03)	0.01
Non-Hispanic white race/ethnicity	2.61 (1.70–4.02)	<0.001		2.76 (1.60–4.76)	<0.001		2.29 (1.11–4.73)	0.03
HIV-positive status	2.72 (1.56–4.77)	<0.001		3.59 (1.86–6.94)	<0.001		1.34 (0.44–4.07)	0.60
Tested by T-SPOT.*TB*	1.58 (1.12–2.24)	0.01		–	–		–	–
Time from diagnosis to treatment	1.00 (1.00–1.01)	0.046		1.00 (1.00–1.01)	0.22		1.01 (1–1.01)	0.03

*aOR, adjusted odds ratio; IGRA, interferon-γ release assay; QFT, QuantiFERON-TB Gold In-Tube; TB, tuberculosis; –, analysis could not be performed. †Multiple logistic models were run by using data from patients having complete data sets for all the included variables; aORs were from the multiple logistic regression models.

**Figure 2:**
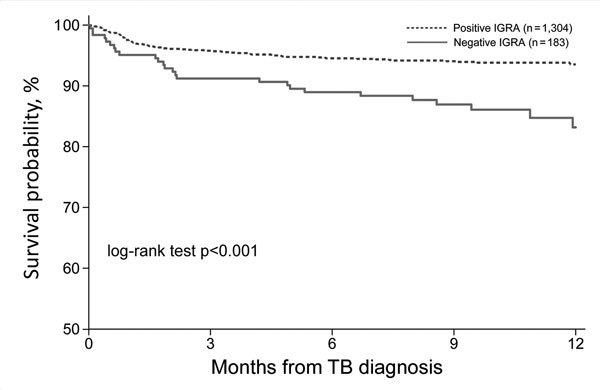
One-year survival from date of TB diagnosis for culture-confirmed TB, stratified by IGRA result, Texas, USA, 2013–2015. IGRA. IGRA, interferon-γ release assay; TB, tuberculosis.

Patients with negative IGRA results had a higher overall mortality than those with positive IGRA results both at the time of diagnosis (2.7% [5/183] vs. 0.7% [9/1,304]; crude OR 4.04, 95% CI 1.34–12.2) and during TB treatment (11.2% [20/178] vs. 4.9% [64/1,295]; crude OR 2.43, 95% CI 1.43–4.13). The 1-year survival rate from diagnosis was 83% for patients with negative IGRA results and 94% for patients with positive IGRA results (p<0.001) (Figure 2), with an adjusted hazard ratio of 1.99 (95% CI 1.18–3.33; p = 0.01) (Table 4). When stratified by IGRA type, only negative T-SPOT.*TB* results remained significantly associated with death. Older age, HIV co-infection, and time to TB treatment were identified by Cox proportional hazards modeling as independent risk factors for death; however, only age remained significant after stratifying by IGRA type (Table 4). The 1-year mortality did not differ significantly by year of TB diagnosis (9.1% for 2013, 6.0% for 2014, and 6.4% for 2015; p = 0.15) (data not shown). Multivariate Cox modeling assessing the survival after the initiation of TB treatment also suggested that negative IGRA results were significantly associated with a higher 1-year mortality (Table 5).

**Table 4:** Cox proportional hazards model for association between 1-year survival from date of tuberculosis diagnosis and characteristics of culture-confirmed TB patients with negative IGRA results, by IGRA type, Texas, USA, 2013–2015*

Variable	All IGRA results, N = 1,343†			QFT only, n = 880†			T-SPOT.*TB* only, n = 463†	
	aHR (95% CI)	p value		aHR (95% CI)	p value		aHR (95% CI)	p value
Negative IGRA result	1.99 (1.18–3.33)	0.01		1.64 (0.88–3.08)	0.120		3.36 (1.27–8.90)	0.02
Age in years	1.06 (1.04–1.07)	<0.001		1.06 (1.04–1.08)	<0.001		1.05 (1.02–1.08)	0.001
Alcohol	1.55 (0.92–2.60)	0.10		1.75 (0.97–3.15)	0.06		1.19 (0.39–3.63)	0.76
HIV positive	5.00 (2.68–9.33)	<0.001		4.79 (2.43–9.47)	<0.001		4.38 (0.90–21.44)	0.07
Time from diagnosis to treatment	0.99 (0.99–1.00)	0.002		0.99 (0.99–1.00)	0.01		0.99 (0.98–1.00)	0.15

*aHR, adjusted hazard ratio; IGRA, interferon-γ release assay; QFT, QuantiFERON-TB Gold In-Tube. †Cox proportional hazards models were run with data from patients having complete data sets for all the included variables; aHRs were from the multivariate Cox proportional hazards models.

**Table 5:** Cox proportional hazards model for association between 1-year survival from tuberculosis treatment start date and characteristics of culture-confirmed TB patients with negative IGRA results, by IGRA type, Texas, USA, 2013–2015*

Variable	All IGRA results, N = 1,343†			QFT only, n = 880†			T-SPOT.*TB* only, n = 463†	
aHR (95% CI)	p value		aHR (95% CI)	p value		aHR (95% CI)	p value
Negative IGRA result	2.12 (1.24–3.63)	0.01		1.84 (0.98–3.47)	0.06		3.41 (1.18–9.88)	0.02
Age in years	1.06 (1.04–1.07)	<0.001		1.06 (1.04–1.07)	<0.001		1.05 (1.02–1.09)	0.002
Alcohol use	1.42 (0.82–2.48)	0.21		1.57 (0.85–2.93)	0.15		1.06 (0.30–3.80)	0.92
HIV positive	4.68 (2.40–9.12)	<0.001		4.60 (2.26–9.34)	<0.001		2.75 (0.32–23.40)	0.36
Time from diagnosis to treatment	0.99 (0.99–1.00)	0.002		0.99 (0.99–1.00)	0.01		0.99 (0.98–1.00)	0.10

*aHR, adjusted hazard ratio; IGRA, interferon-γ release assay; QFT, QuantiFERON-TB Gold In-Tube; TB, tuberculosis. †Cox proportional hazards models were run with data from patients having complete data sets for all the included variables; aHRs were from the multivariate Cox proportional hazards models.

## Discussion

Our results suggest that TB patients with false-negative IGRA results had significantly poorer outcomes, with nearly 2 times the odds for death within 1 year of TB diagnosis, compared with patients with positive IGRA results. False-negative IGRA results were more likely to occur in older, non-Hispanic white, and HIV-infected patients or patients tested with the T-SPOT.*TB* assay.

Our study not only confirms the World Health Organization and Centers for Disease Control and Prevention recommendation of not using IGRAs as rule-out tests for TB disease (*7**,**16*) but also indicates that patients with confirmed TB diagnoses but negative IGRA results have poor outcomes. Our study results highlight the need for having a systematic and extensive management strategy for suspected TB patients who have negative IGRA results to minimize misdiagnosis and improve patient outcomes. In symptomatic patients being evaluated for TB disease, further diagnostic evaluation and close follow-up for TB disease should be considered, especially in persons >60 years of age, non-Hispanic white, HIV-infected, or tested by the T-SPOT.*TB* assay.

Although false-negative IGRA results were associated with a nearly 2-fold increase in risk for death within 1 year of TB diagnosis, only a false-negative T-SPOT.*TB* result remained significantly associated with death after stratification by IGRA type. This finding suggests that patients who had false-negative T-SPOT.*TB* results in our study might be sicker than those who had false-negative QFT results. This explanation is consistent with the significant delay in treatment initiation after TB diagnosis, especially for patients in the T-SPOT.*TB* group. The significant association between time to treatment and death among all patients and patients by IGRA type suggests that delayed treatment might contribute to the higher mortality of patients with false-negative IGRA results. This finding demonstrates the programmatic implications for treating patients who have negative IGRA findings. Consistent with previously published data, we also found a significantly higher risk for death among patients of older age or with HIV co-infection (*17**–**19*).

Consistent with results of previous studies (*8**–**10**,**20*), in our study, older age was also significantly associated with a false-negative result for both the QFT and T-SPOT.*TB* assays. An explanation for this association could be the gradual decrease of IFN-γ production that occurs in response to *M. tuberculosis*–specific antigens ESAT-6 and CFP-10 with age (*9**,**10*). Other authors have observed a higher rate of false-negative IGRA results in young children (*21**–**23*). However, all 17 children <5 years of age in our study had positive IGRA results.

HIV co-infection was associated with a false-negative QFT result but not a false-negative T-SPOT.*TB* result. This finding is consistent with the current literature, which has suggested that the T-SPOT.*TB* assay has higher sensitivity than QFT among HIV co-infected persons, especially those with low CD4+ T-cell counts (*24**,**25*). As observed in a previous study (*26*), in our study, non-Hispanic white race/ethnicity was also associated with a false-negative QFT result. In our analysis, extrapulmonary TB was not significantly associated with a false-negative IGRA result or patient outcomes.

Our study has several limitations. We could not completely rule out some provider selection bias, considering nearly 48% (1,367/2,854) of the culture-confirmed TB patients were excluded from the analysis, most (94%, 1,284/1,367) because an IGRA was not performed. Because we used deidentified administrative data obtained from NEDSS, quantitative test results for QFT and T-SPOT.*TB* were not available for analytic evaluation. In addition, by selecting only nonindeterminate results (for QFT) and nonfailed results (for T-SPOT.*TB*), the IGRAs’ true sensitivities were overestimated (89.0% [868/975] for QFT and 85.2% [436/512] for T-SPOT.*TB*; p = 0.031). If indeterminate and failed results are included with the negative results when measuring the sensitivity, then the true sensitivity of the QFT and TSPOT.*TB* assays in this population-based screening becomes 84.4% for QFT and 80.9% for T-SPOT.*TB*, and the difference in the sensitivity between QFT and T-SPOT.*TB* becomes nonsignificant (p = 0.095). Immunologic and genetic details, which can help explain the cause of false-negative IGRA results, were also unavailable. Last, because some of the data were originally obtained from interviewing TB patients, the possibility of recall bias cannot be ruled out.

In conclusion, we identified negative IGRA results as a significant predictor for death within 1 year of TB diagnosis among culture-confirmed *M. tuberculosis*–infected patients. Older age, non-Hispanic white race/ethnicity, HIV co-infection, T-SPOT.*TB* assay results, and longer time to TB treatment were significantly associated with false-negative IGRA results. Healthcare providers should consider these risk factors when making decisions on whether to initiate further diagnostic evaluations for TB patients with negative IGRA results.

Technical Appendix Characteristics of included and excluded culture-positive tuberculosis patients in study of characteristics associated with false-negative interferon-γ release assay results.
