# Supplementary material for: Characteristics Associated with Negative Interferon-γ Release Assay Results in Culture-Confirmed Tuberculosis Patients, Texas, USA, 2013–2015
Source: Emerg Infect Dis. 2018 Mar;24(3):534–40. doi: 10.3201/eid2403.171633 (PMC5823348; doi:10.3201/eid2403.171633)
Supplement: Technical Appendix — Characteristics of included and excluded culture-positive tuberculosis patients in study of characteristics associated with false-negative interferon-γ release assay results. [file 17-1633-Techapp-s1.pdf]

Article DOI: https://doi.org/10.3201/eid2403.171633

Characteristics Associated with Negative Interferon-γ Release Assay Results in Culture-Confirmed Tuberculosis Patients, Texas, USA, 2013–2015

Technical Appendix

Technical Appendix Table. Demographic and clinical characteristics of included and excluded populations in study of characteristics associated with negative IGRA results in culture-confirmed TB patients, Texas, USA, 2013–2015*

Variable	Excluded, N = 1,367	Included, N = 1,487	p value
Age, y, median (IQR)	48 (31–60)	47 (30–61)	0.733
Age ≥60 y	354 (25.9)	402 (27.0)	0.490
Sex			
M	956 (69.9)	942 (63.3)	<0.001
Race/Ethnicity			
Non-Hispanic white	167 (12.2)	152 (10.2)	0.091
Black	251 (18.4)	275 (18.5)	0.927
Hispanic	737 (53.9)	762 (51.2)	0.154
Asian	207 (15.1)	288 (19.4)	0.003
Other	5 (0.4)	10 (0.7)	0.258
Foreign born	747 (54.6)	897 (60.3)	0.002
Resident of long-term care facility	19 (1.4)	20 (1.3)	0.918
Homeless	98 (7.2)	100 (6.7)	0.641
Excess alcohol use	283 (20.7)	257 (17.3)	0.019
Injection drug use	60 (4.4)	38 (2.6)	0.007
TB-CXR	1,186 (91.9)	1,240 (88.6)	0.004
HIV positive	88 (7.5)	90 (6.6)	0.358
Multidrug resistance	8 (0.6)	13 (0.9)	0.367
Final vital status, dead	182 (13.3)	107 (7.2)	<0.001

*Values are no. (%) unless otherwise specified. IGRA, interferon-γ release assay; IQR, interquartile range; TB, tuberculosis; TB-CXR, tuberculosis-specific abnormalities on chest radiograph.
